# Purification, and characterization of xylanase by *Aspergillus krugeri* AUMC 15912 utilizing sugarcane bagasse under solid-state fermentation conditions

**DOI:** 10.1186/s12866-026-04742-0

**Published:** 2026-02-09

**Authors:** Aya A. A. Abdel-Hafeez, Abdel-Raouf M. Khallil, Elhagag A. Hassan, Osama A. M. Al-Bedak

**Affiliations:** 1 Department of Industrial Biotechnology, Faculty of Sugar and Integrated Industries Technology, Assiut University, Assiut, 71511 Egypt; 2 Department of Botany and Microbiology, Faculty of Science, Assiut University, Assiut, Egypt; 3 Assiut University Mycological Centre, Assiut, Egypt; 4 ERU Science & Innovation Center of Excellence, Egyptian Russian University, Badr City, Cairo 11829 Egypt

**Keywords:** Aspergillus, Bagasse, Enzymes, Lignocellulose, Xylanase, Waste

## Abstract

This study focuses on optimizing, purifying, and characterizing xylanase produced by *Aspergillus krugeri* AUMC 15912 utilizing sugarcane bagasse under solid-state fermentation (SSF). The strain, isolated from sugarcane bagasse, exhibited significant xylanase activity, which was maximized after six days of incubation at pH 7.0 and 30 °C using ammonium chloride as the nitrogen source, yielding 183 ± 16 U/g dry substrate (gds). The enzyme was purified 508-fold through sequential chromatography on Trilite MC 08 and Sephacryl S-200 columns, resulting in a single band of 33.11 kDa on SDS-PAGE. The purified xylanase displayed peak activity of 1697.5 U/mg at pH 6.0 and 45 °C, with kinetic parameters Km and Vmax of 1.75 mM and 17.98 µmol/min, respectively. Activity was influenced by various salts and inhibitors, with residual activity ranging from 30.12% (NiCl₂) to 86.65% (CuSO₄). These findings highlight the potential of *A. krugeri* AUMC 15912-derived xylanase for industrial applications in bioconversion, food processing, and biofuel production.

## Introduction

 The primary byproduct of the sugarcane manufacturing process is sugarcane bagasse, which consists of approximately 50% cellulose, 25% hemicelluloses, and 25% lignin [1]. Consequently, the recycling and repurposing of such waste are increasingly challenging. The fibrous structure of sugarcane bagasse, coupled with its high concentrations of cellulose and hemicellulose, stimulated microorganisms to generate xylanase, and additionally, electrical power, sugars, enzymes, second-generation biofuels (ethanol), and other value-added products [2]. Due to its widespread availability, it serves as the optimal substrate for microbial fermentations that yield various value-added products [3, 4]. Several fungal species, including *Aspergillus*, *Fusarium*, *Penicillium*, and *Rhizopus*, have been identified for their capacity to generate xylanase from cost-effective hemicellulosic agricultural byproducts, such as sugarcane bagasse, rice straw, rice bran, wheat straw, and corn cob [5, 6].

Lignocellulose is a viable, renewable supply of raw materials for bio-based compounds (including polymers and xylooligosaccharides) and biofuels (such as bioethanol and biodiesel), all of which are in high demand [7]. The composition mostly comprises 40–50% cellulose, 25–30% hemicellulose, and 15–20% lignin, which together form the complex and robust cross-linked structure of plant cell wall biomass [8]. Xylan, the main constituent of hemicellulose and the second most prevalent polysaccharide in nature [9], consists of 1,4-β-linked D-xylose units [10–13].

Xylanases hydrolyze the β−1,4 D-xylopyranosyl links of β−1,4 D-xylans, resulting in the formation of monomeric and oligomeric sugars [14]. The promise of xylanases in various sectors, including food, paper, pulp, textiles, animal feed, and biofuels, has prompted significant research efforts [5]. Microbial xylanases have demonstrated significant biotechnological potential across various sectors beyond bioenergy production, including enhancing dough elasticity in food, promoting chick weight in animal husbandry, clarifying juices for fiber degumming, manufacturing bread, prebleaching Kraft pulp, deinking recycled newspapers, and winemaking [15]. *Aspergillus* and other filamentous fungi are considered efficient suppliers of hydrolytic enzymes for holocellulose breakdown due to their capacity to swiftly adapt to solid substrates by emulating their native habitat with lignocelluloses [16]. Cost-efficient enzymes are essential for effective functioning of biotechnological applications. Consequently, using pure xylan as a stimulant for enhanced xylanase production is impractical. The use of lignocellulosic waste offers cost-effective substrates and beneficial environmental impacts, representing a notable advancement in this domain. The utilization of agro-residues for microbial enzyme production has progressed markedly [6, 17, 18].

In addition to its application in the pulp and paper sector, xylanases serve as food additives [19], enhance dough handling and the quality of baked goods in wheat flour [20, 21], facilitate the extraction of coffee, plant oils, and starch [22], improve the nutritional properties of agricultural silage and grain feed [22], and, when combined with pectinase and cellulase, assist in the clarification of fruit juices [23].

Following the extraction of sugarcane juice, a fibrous residue known as sugarcane bagasse is produced. It functions as a crucial source of cellulose and hemicellulose for biofuel generation [24]. The principal constituents of sugarcane bagasse are 42–58.2% cellulose, 9.2–25% hemicellulose, and 13.4–20% lignin [25, 26], which might serve as a byproduct for the production of these biopolymers applicable in many fields. Approximately 700 million tons of sugarcane bagasse are generated globally each year [27, 28]. *A. krugeri* AUMC 15912 was utilized to enhance xylanase output in submerged fermentation (SmF). Solid-state fermentation (SSF) was utilized to generate an economical xylanase by *A. krugeri* AUMC 15912, employing sugarcane bagasse as the substrate. Subsequently, the xylanase enzyme was subjected to purification and characterization.

## Materials and methods

### Fungal strain and its Xylanase activity

This investigation utilized a strain, belonging to the genus *Aspergillus*, that obtained from sugarcane bagasse by the direct plating method [29]. Five segments of sugarcane bagasse were cultured on Petri dishes containing potato dextrose agar (PDA; [30]. The plates were thereafter incubated at 28 °C for 10 days until fungal colonies developed. The proliferating fungi were isolated on PDA and preserved as frozen cultures at −86 °C, as well as on cotton balls [31]. In an ordinary evaluation of xylanase activity utilizing sucrose-free Czapek’s agar enriched with 1.0% oat spelts xylan, the strain was identified as a high xylanase producer.

### Morphological and molecular identification of the potent strain

To morphologically identify the *Aspergillus* strain, the fungus was inoculated onto Czapek’s agar (CzA), Malt Extract Agar (MEA), and Czapek’s Yeast Autolysate Agar (CYA) (Smith and Onions 1994), and cultured for 7 days at 25 °C. The fungus in this research was morphologically characterized based on its macroscopic and microscopic traits [32]. This strain was deposited and assigned the accession number AUMC 15912 in the culture collection of the Assiut University Mycological Centre, Assiut, Egypt. DNA isolation was conducted [33], and the PCR reaction was executed by SolGent Co., Ltd (Daejeon, South Korea) utilizing SolGent EF-Taq and the universal primers ITS1 and ITS4 [34]. DNASTAR (version 5.05) was employed to produce the contiguous sequences of the *Aspergillus* strain in this study. The most similar sequences, from the genus *Aspergillus*: section Flavi, to the strain in this investigation were sourced from GenBank. All sequences were aligned using MAFFT (version 6.861b) with default settings [35]. The alignment gaps and sparse uninformative characters were optimized using BMGE [36]. Phylogenetic studies utilizing maximum-likelihood (ML) and maximum-parsimony (MP) methods were conducted with MEGA X (version 10.2.6) [37], incorporating 1000 replications [38] to evaluate the robustness of the most parsimonious tree. The optimal nucleotide substitution model for maximum likelihood analysis was determined using the Akaike Information Criterion (AIC) in Modeltest 3.7 [39]. Subsequent to alteration, the tree was preserved in TIF format.

### Improvement of Xylanase production in SSF

To maximize xylanase production in SSF, the pH (3.0, 4.0, 5.0, 6.0, 7.0, 8.0, 9.0, and 10.0), nitrogen sources (peptone, yeast extract, beef extract, sodium nitrate, ammonium sulphate, and ammonium chloride, each at 0.2%), temperatures (25, 30, 35, and 40 °C), and incubation durations (1–8 days) for *A. krugeri* AUMC 15912 were systematically altered under one-factor-at-a-time (OFAT) conditions. The experiment was performed in 500 mL Erlenmeyer flasks, each containing 10 g of sugarcane bagasse as substrate. The substrate was individually hydrated with 10 mL of sucrose-free Cz broth [40] supplemented with 0.1% oat spelt xylan solution. The moisture content was modified to 80% by incorporating a buffer solution into each flask. After autoclaving, the flasks were separately inoculated with a 5.0 mL spore suspension containing 1.5 × 10^8^ spores/mL, derived from a 7-day-old culture of *A. krugeri* AUMC 15912. Independent replicates for each sampling point were conducted using three distinct flasks for each sampling day. Enzymatic activity was assessed every day in triplicate.

### Xylanase assay and protein determination

To measure the xylanase activity, 0.5 mL of filtered crude enzyme was mixed with 0.5 mL of 1.0% oat spelt xylan, which was made in a 50 mM sodium citrate buffer (pH 5.0). After 15 min of incubation in water bath at 50 °C, the reaction was stopped by adding 2 mL of 3,5-dinitrosalicylic acid (DNS), and boiling the mixture for 10 min [17, 41]. After cooling, a UV-Visible spectrophotometer (T80+, Manchester, UK) was used to quantify the color absorbance at 540 nm. Using the xylose standard curve, the amount of reducing sugar released was determined. The amount of xylanase enzyme that causes the release of one µmol of xylose equivalent per milliliter under standard assay conditions is called one unit of xylanase. The total protein concentration was determined following the procedure outlined by [42]. The cell-free supernatant was extracted by centrifugation (10,000 rpm for 15 min at 4 °C), and cold 100% ethyl alcohol (−25 °C) was slowly added to the clear supernatant while it was being gently agitated at 4 °C. The total protein was then isolated, lyophilized, and subsequently used in purification process.

### Purification of the Xylanase produced by *A. krugeri* AUMC 15912

#### Trilite MC 08 cation exchange column

Trilite MC 08 cation exchange resin with a bed capacity of 400 cm³ was activated by 5% HCl and positioned within a glass column of 100 cm in height and 5.0 cm in diameter. Twenty milliliters of the crude xylanase sample were applied to the ion exchange gel. The bound proteins were eluted utilizing a citrate buffer (100 mM, pH 5.0) and a gradient of NaCl concentrations from 0 to 1.5 M, at a flow rate of 0.5 mL/min. Protein content and xylanase activity measurements were conducted on collected 5.0 mL fractions. Xylanase assay was conducted in all collected fractions. The reaction mixture comprised 0.5 mL enzyme filtrate and 0.5 mL of 1% oat spelt xylan (pH 6.0). The reaction mixture was incubated for 10 min at 45 C. The abovementioned method of Miller [41] was followed to determine the reducing sugars. The most active fractions were pooled, concentrated, and utilized in additional purification processes.

#### Sephacryl S 200 size exclusion column

After the concentrated xylanase sample was buffered with citrate buffer (100 mM, pH 5.0), it underwent further purification using a Sephacryl S 200 size exclusion column (50 cm × 2.4 cm) with a bed capacity of 100 cm³. Five milliliters of the xylanase sample were applied to the gel. Maintaining a flow rate of 0.25 mL/min, the protein was eluted using 100 mM citrate buffer (pH 5.0). The characterization experiments made use of the pooled and lyophilized fractions that were determined to be the most active.

### Determination of Xylanase molecular weight by SDS-PAGE

An RIPA lysis buffer containing 1 µg/mL of leupeptin and aprotinin, 0.5 mM of phenylmethylsulphonyl fluoride (PMSF), 1 mM of sodium hydroxide, and 5 mM of sodium fluoride was used to dissolve protein samples, whether they were crude extracts or purified. The buffer also included 50 mM Tris-HCl (pH 7.6), 5 mM EDTA, 150 mM sodium chloride, 0.5% of NP-40, and 0.5% of Triton-X-100. After estimating protein concentrations using the Lowry method [42], sample buffer was added to portions of protein samples and boiled for 5 min. In the wells, along with 5 µL of pre-stained protein marker (BLUtra prestained protein ladder; GeneDireX; Taiwan), 30 µL of sample (representing nearly 30 µg/mL of protein) were added. The stacking gel was electrophoresed at 60 V and the separating gel at 120 V while the gel was cooled. The gel was removed after electrophoresis and stained with Coomassie blue for 30 min. The staining was left overnight.

### Effect of pH, temperature, and some chemicals on activity of the pure Xylanase

The activity of pure xylanase was assayed at pH values between 3 and 10 and temperatures ranging from 30 to 55 °C. The enzyme powder and oat spelt xylan (0.01 g each) were separately dissolved in 1.0 mL of a 50 mM buffer solution in the reaction mixture. After the 5-minute reaction time was over, the xylanase activity was measured in the same way as before. Also, the effect of some chemicals such as NaCl, KCl, CaCl_2_, MgSO_4_, MnSO_4_, ZnSO_4_, FeSO_4_, CuSO_4_, and NiSO_4_, Sodium dodecyl sulfate (SDS), and ethylenediaminetetraacetic acid (EDTA) each at a concentration of 5 mM, was evaluated. The experiment was repeated three times.

### Determination of Km and vmax

Using a pure oat spelts xylan experiment with substrate concentrations ranging from 1 to 10 mg/mL, the kinetic constants–Km and Vmax–for xylanase activity were determined [43].

### Statistical analysis

After conducting the initial investigation three times, the mean and standard deviation (SD) were used to express all data. The statistical significance analysis was conducted [44], and the significance level was set at *p* ≤ 0.05.

## Results

### Morphological and molecular identification of the potent strain

**Fig. 1:**
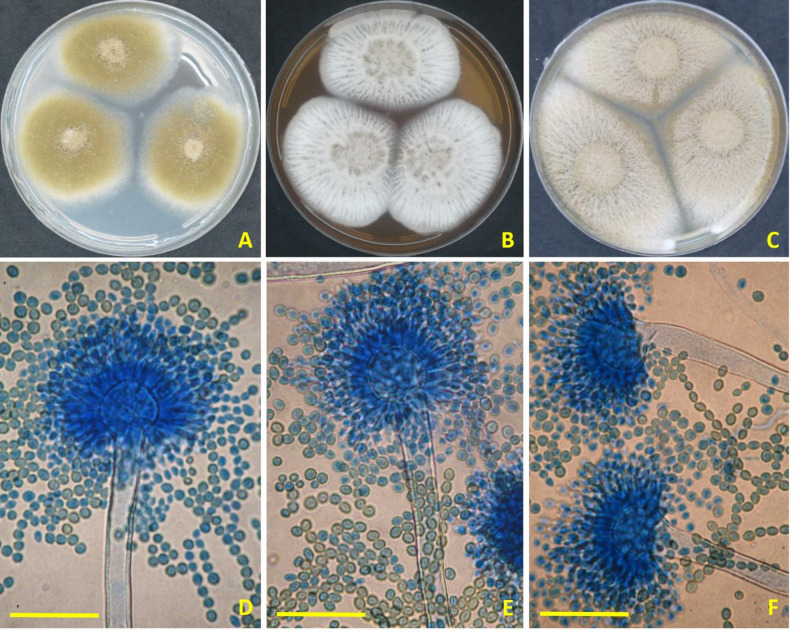
*Aspergillus krugeri* AUMC 15912 (**A**–**C**) Seven-day-old colonies on Cz, MEA, and CYA at 25 °C (**D**–**F**) Biserriate conidial heads and conidia (Scale bars = 100 μm)

The strain used in this investigation was highly like species of *A. krugeri* (type species). After 7 days at 25 °C, colonies on CYA granular and velutinous with white mycelial patches and moderate to dense sporulation. Conidial heads radiate. Conidiophores rough, hyaline 350–1000(–1300) × 10–18(–21) µm. Vesicles globose or spathulate, 40–80 μm. Metulae 11–22.5 μm. Phialides ampulliform, 10–15 × 4.5–7 μm. The ascomata are not visible. Conidia rough, broadly ellipsoid 4–7 × 3.5–6.5 μm (Fig. 1).

**Fig. 2:**
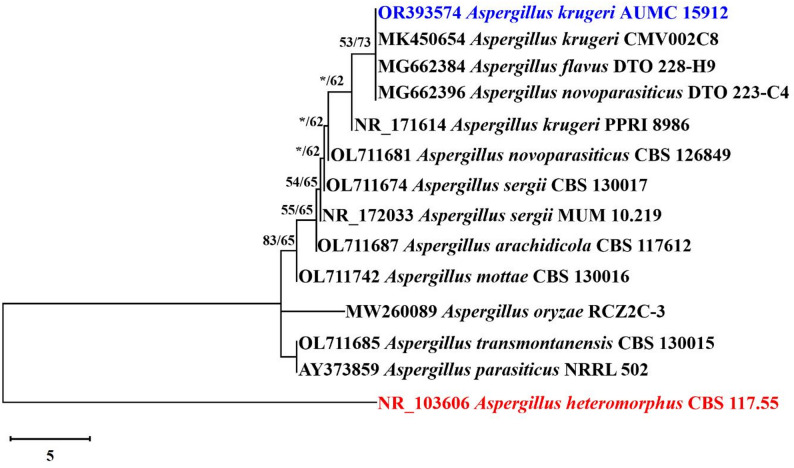
Maximum parsimonious phylogenetic tree produced by ML/MP analysis of *A. krugeri* strain AUMC 15912’ ITS sequence in this work (in blue) in comparison to sequences of the *A. flavus* group’s most closely related species in GenBank. Near the corresponding nodes are the bootstraps (1000 replications) with ML/MP support values ≥ 50%. *Aspergillus heteromorphus* CBS 117.55 is used as the tree root (in red)

The strain identification was confirmed using phylogenetic analysis based on ITS sequencing. Out of the 884 characters in the final ITS data set, 14 sequences were used to generate them; 554 of those characters could be aligned successfully, 43 were considered variable, and 2 were informative. The ideal model for representing the link between taxa was the Tamura 3-parameter (T92) model. The most parsimonious tree (out of 9) that was generated using the Maximum Parsimony approach had a tree length of 52 steps, a greatest log likelihood of −1491.12, a consistency index of 0.857143, a retention index of 0.900000, and a composite index of 0.771429 (Fig. 2).

### Improvement of xylanase production in SSF

**Fig. 3:**
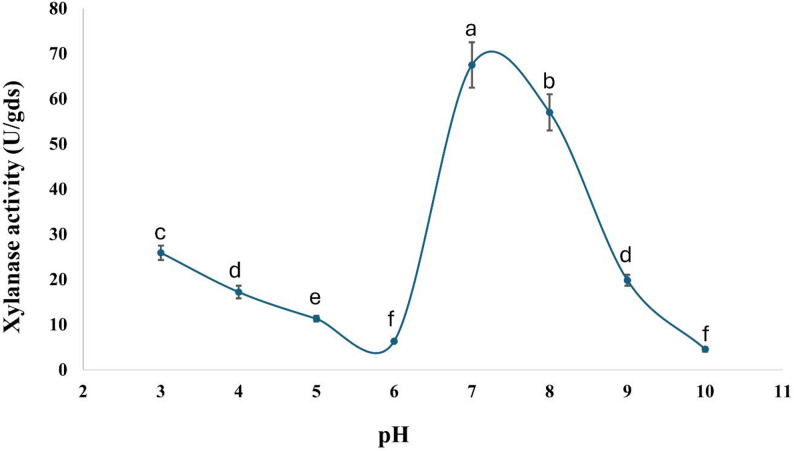
Effect of medium’s pH on xylanase production by *A. krugeri* AUMC 15912 under SSF (Mean values ± SD on with different letters are significantly different; *p* < 0.05; *n* = 3)

**Fig. 4:**
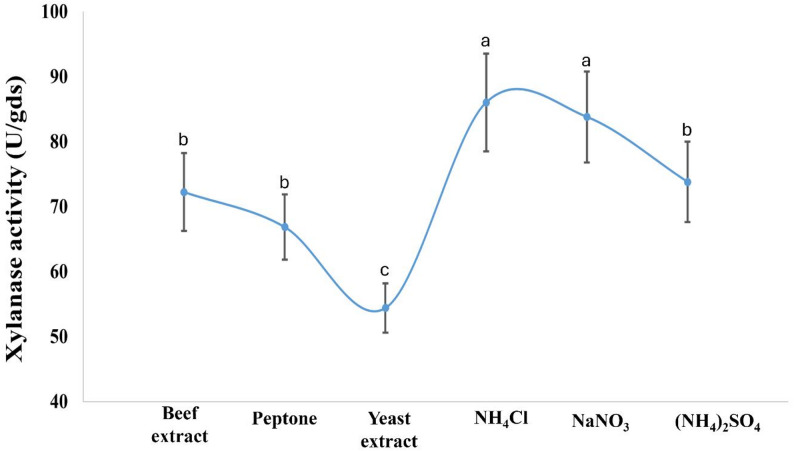
Effect of medium’s nitrogen source on xylanase production by *A. krugeri* AUMC 15912 under SSF (Mean values ± SD on with different letters are significantly different; *p* < 0.05; *n* = 3)

**Fig. 5:**
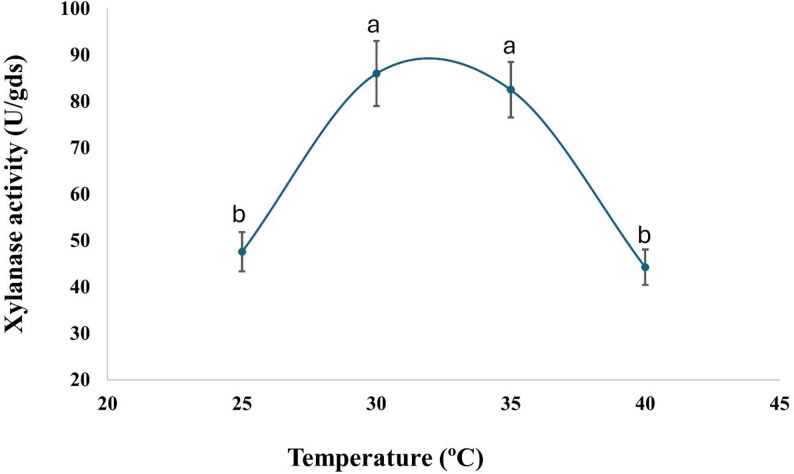
Effect of incubation temperature on xylanase production by *A. krugeri* AUMC 15912 under SSF (Mean values ± SD on with different letters are significantly different; *p* < 0.05; *n* = 3)

**Fig. 6:**
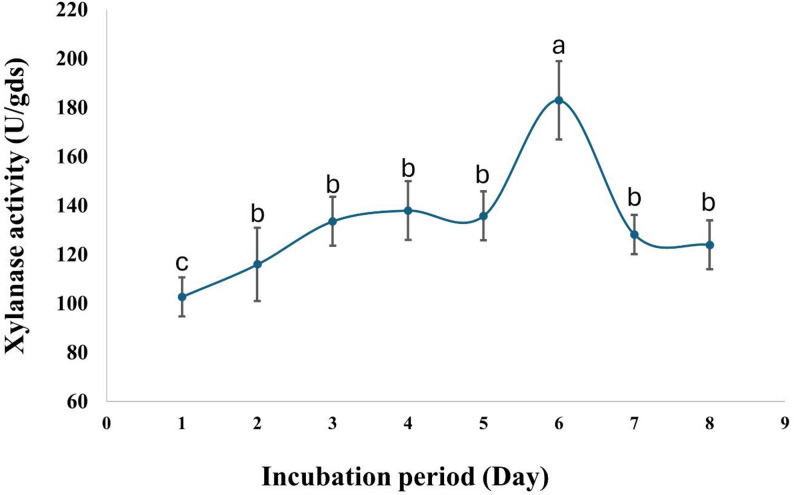
Effect of incubation period on xylanase production by *A. krugeri* AUMC 15912 under SSF (Mean values ± SD on with different letters are significantly different; *p* < 0.05; *n* = 3)

The current findings indicate that *A. krugeri* AUMC 15912 exhibited xylanase activity of 67.5 ± 5 U/gds at pH 7.0 (Fig. 3). The addition of ammonium chloride as a nitrogen source to the fermentation medium resulted in an increase of xylanase activity to 86.0 ± 7 U/gds at 30 °C (Figs. 4 and 5). The peak xylanase activity occurred after six days of incubation, resulting in 183 ± 16 U/gds (Fig. 6).

### Purification of xylanase

**Fig. 7:**
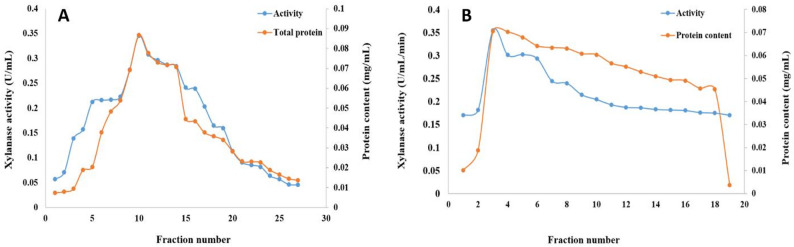
Diagrams of xylanase purification using (**A**) Trilite MC 08 cation exchange column (**B**) Sephacryl S 200 size exclusion column chromatography

The fermentation of sugarcane bagasse for six days, utilizing ammonium chloride as a nitrogen source at pH 7.0 and 30 °C, enabled *A. krugeri* AUMC 15912 to produce xylanase under solid-state fermentation conditions. The total protein was extracted by precipitating the cell-free supernatant (2000 mL) using 70% saturated ammonium sulfate. The dialyzed protein was subjected to precipitation and lyophilization. Trilite MC 08 column yielded 7 pooled fractions (Fig. 7A), whereas Sephacryl S 200 generated 16 active fractions with xylanase and protein peaks (Fig. 7B).

Following processing in two columns, the specific activity of the purified xylanase was 508 times higher, yielding 14.73% protein and 1697.5 U/mg of xylanase activity. Table 1 provides a summary of the steps used to purify the xylanase.

**Table 1:** Purification profile of the Xylanase produced by *A. krugeri* AUMC 15912 at pH 7.0 and 30 °C after 6 days using ammonium chloride as nitrogen supply in SSF

Purification steps	Volume (mL)	Activity (U/mL)	Total activity (U)	Protein (mg/mL)	Total protein (mg)	Specific Activity (U/mg)	Yield (%)	Fold
Fermentation medium	2000	1.89	3780	0.566	1132	3.34	100	1
Ammonium sulphate	80	23.3	1864	3.2	256	7.28	49.3	2.18
Trilite MC 08	50	14.62	731	0.67	33.5	21.82	19.34	6.53
Sephacryl S 200	20	27.84	556.8	0.0164	0.328	1697.5	14.73	508

### SDS-PAGE

**Fig. 8:**
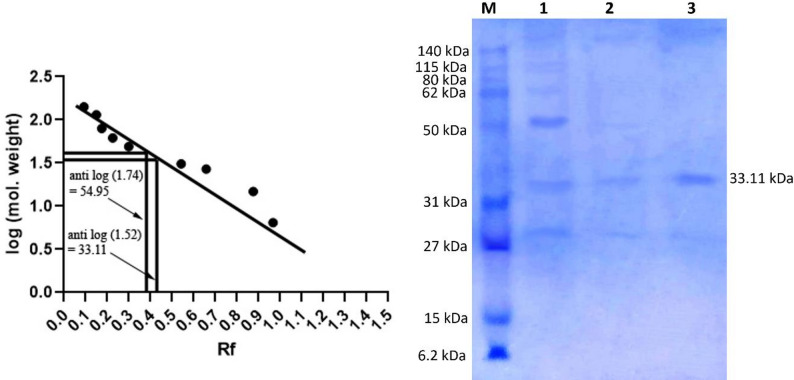
SDS-PAGE of purified xylanase produced by *A. krugeri* AUMC 15912. M: Pre-stained protein marker, Lane 1: Crude xylanase. Lane 2: Xylanases from Trilite MC 08 column. Lane 3: Xylanase from Sephacryl S 200 column

This study’s xylanase was purified to a single band with a molecular weight of 33.11 kDa, according to SDS-PAGE data (Fig. 8).

### Effect of pH and temperature on the activity of the pure xylanase

**Fig. 9:**
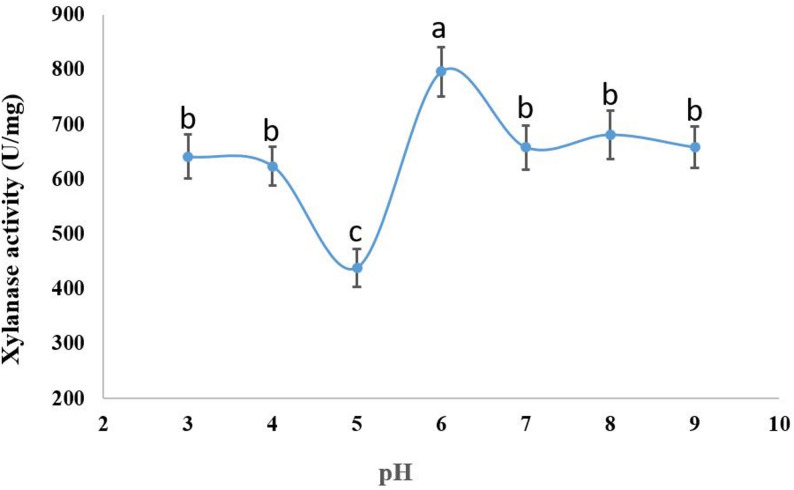
Effect of pH on the activity of the pure xylanase produced by *A. krugeri* AUMC 15912 under SSF (Mean values ± SD on with different letters are significantly different; *p* < 0.05; *n* = 3)

**Fig. 10:**
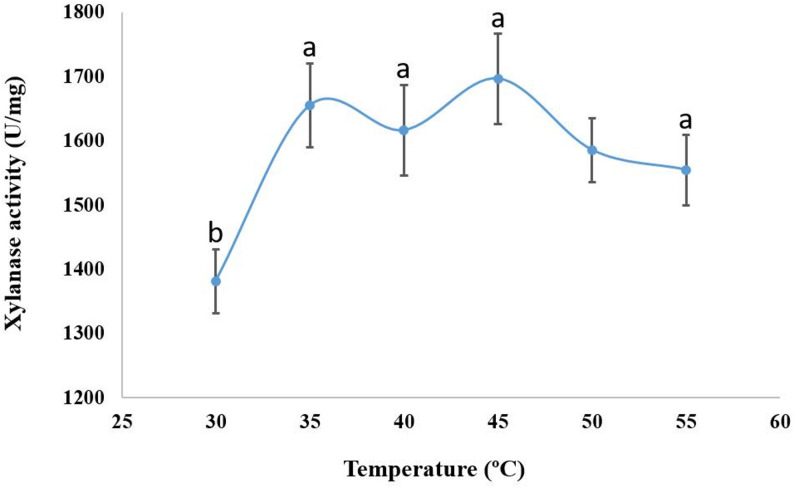
Effect of temperature on the activity of the pure xylanase produced by *A. krugeri* AUMC 15912 under SSF (Mean values ± SD on with different letters are significantly different; *p* < 0.05; *n* = 3)

The xylanase activity was tested at 50 °C with varying pH levels (3–10). Xylanase activity, which yielded 796.5 ± 45 U/mg of enzyme activity, was best achieved at a pH of 6.0 (Fig. 9). The purified xylanase exhibited activity across a broad temperature range of 30 to 55 °C. At 45 °C, the xylanase activity reached its maximum of 1697.5 ± 90 U/mg, whereas the enzyme maintained 81.4%, 97.5%, 95%, 93.4%, and 91.6% of its activity at 30, 35, 40, 50, and 55 °C, respectively (Fig. 10).

### Effect of some chemicals on xylanase activity

At the optimum conditions of pH 6.0 and 45 °C, the effect of some chemicals on the pure xylanase was evaluated. From 30.12% inhibition for NiCl_2_ (511 ± 15 U/mg) to 86.65% for CuSO_4_ (1470 ± 60 U/mg), the xylanase specific activity was reduced to different degrees by the investigated compounds (Table 2).

**Table 2:** Effect of various ions and inhibitors (at pH 6.0 and 45 °C) on the activity of the pure Xylanase produced by *A. krugeri* AUMC 15912 (The results are means of three replicates ± SD; figures in the table with different letters are significantly different at *p* < 0.05)

Chemicals	Specific activity (U/mg)	Residual activity (%)
Control	1696.5 ± 90^a^	100
NaCl	623.7 ± 22^f^	36.76
KCl	773.7 ± 42^e^	45.6
CaCl_2_	1259 ± 35^c^	74.2
MgSO_4_	1467 ± 65^d^	86.47
MnSO_4_	927 ± 70^d^	54.64
ZnSO_4_	1555 ± 70^b^	91.68
CuSO_4_	1470 ± 60^b^	86.65
FeSO_4_	970 ± 50^d^	57.17
NiCl_2_	511 ± 15^g^	30.12
EDTA	681.5 ± 25^f^	40.17
SDS	962.6 ± 35^d^	56.74

### Determination of Km and vmax

**Fig. 11:**
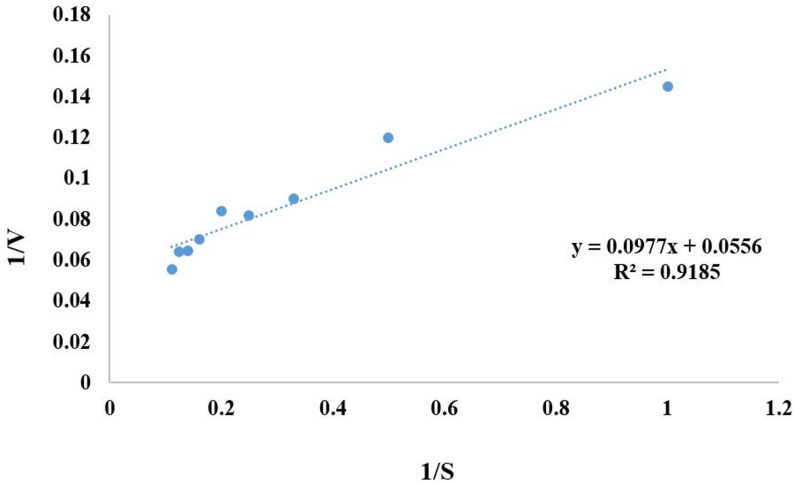
Lineweaver-Burk plot of the reciprocal of initial velocity and xylan concentration

The current results demonstrated that Km and Vmax for the purified xylanase were determined to be 1.75 mM and 17.98 µM/min, respectively (Fig. 11).

## Discussion

The high cost of enzyme manufacturing necessitates the identification of an economical growing medium for microorganisms to satisfy industrial enzyme demand. Xylanases are required in substantial amounts for industrial applications because to their versatility and resilience to the severe conditions present in these processes. Consequently, it is essential to select microorganisms that exhibit the requisite characteristics for the prolific synthesis of xylanases, and subsequently adjust cultivation conditions to enhance enzyme yield [40, 45]. Both the medium’s nutritional composition and the culture conditions have a substantial impact on xylanase output.

To maximize xylanase production, the *A. krugeri* AUMC 15912 used in this study had its culture parameters modified for SSF, including pH, nitrogen supply, temperature, and incubation period. The current findings showed that *A. krugeri* AUMC 15912 achieved a peak of xylanase activity of 183 ± 16 U/gds after incubating for six days at pH 7.0 and 30 °C with ammonium chloride as the nitrogen source. It has been shown that xylanase production is affected by physical and chemical parameters such as pH, temperature, incubation period, and concentrations of carbon and nitrogen [40, 46]. Studies have shown that multiple fungal and bacterial species have been utilized for xylanase production. Xylanase was generated from wheat bran at quantities between 191 and 738 U/gds by several strains of *Aspergillus flavus* under solid-state fermentation conditions [47]. During solid-state fermentation, *Aspergillus oryzae* increased the amount of xylanase it produced from wheat bran to 2830.7% U/gds [48]. *A. flavus* produced 32.4 IU/gds in total [49]. Solid-state fermentation of oil palm empty fruit bunches by *A. niger* USM SD2 achieved a maximal xylanase activity of 3246 IU/gds at pH 6.3 and 25 °C, utilizing yeast extract as a nitrogen source [50]. Using palm kernel cake and palm pressed fiber, *A. niger*, *A. oryzae*, and *A. awamori* produced xylanase at concentrations of 18.8, 27.2, and 134.2 U/gds, respectively [51]. After fermenting the discarded fruit bunches, *A. fumigatus* ITBCCL170 produced 236.30 U/g xylanase [52]. The maximum xylanase activity (2210.23 ± 138.50 U/gds) was produced by *A. niger* CECT 2700 using brewery spent grain after 5 days of fermentation [53]. It is not always easy to compare enzyme activity values across different research because of small methodological differences. Therefore, it is important to exercise caution when drawing analogies.

The xylanase in this study was purified 508-fold using two columns, resulting in the isolation of a single molecular weight band of 33.11 kDa. At a pH of 6.0 and a temperature of 45 °C, the xylanase exhibited peak activity of 1697.5 ± 90 U/mg. The optimal pH range for xylanases sourced from *Aspergillus* species typically lies between pH 3.0 and 6.0 [54]. The results were consistent with those reported by Ameen (2023), who showed that the xylanase activity of *A. fumigatus* KSA-2 peaked at pH 6.0 and 45 °C. The present findings corroborate previous studies suggesting that the ideal pH for xylanase produced by *A. fumigatus* SK1 is pH 4.0 [55], while activity peak of xylanase produced by *A. oryzae* LC1 was established at pH 5.0 [56].

This research investigated the influence of ions and inhibitors on xylanase activity. The effect of several substances on pure xylanase was evaluated under ideal conditions of pH 6.0 and 45 °C. The specific activity of xylanase was reduced to different degrees by the tested compounds, with inhibition ranging from 30.12 ± 0.88% for NiCl2 (511 ± 15 U/mg) to 86.65 ± 3.53% for CuSO4 (1470 ± 60 U/mg). Enzymes can engage with ionic species present in the reaction media. Ions can function as co-factors, either augmenting or suppressing enzymatic activity by creating inert complexes with the enzyme [57]. Evidence indicates that xylanase generated by *Penicillium roquefortii* exhibits effects comparable to those of a commercially available xylanase. Ionic species impeded enzyme activity when present in the reaction medium. The enzymatic activity of xylanase, derived from the solid-state fermentation of yellow mombin residue, augmented by 40% upon the addition of Mn^2+^, whereas it diminished by 50% with the introduction of Cu^2+^ [58]. Xylanase from *A. fumigatus* KSA-2 exhibited a 10.2% and a 128.0% enhancement in specific activity when exposed to Cu^2+^ and Mn^2+^ ions, respectively [40].

## Conclusions

Using sugarcane bagasse as a substrate, this study examined the synthesis of xylanase for the first time using *A. krugeri* AUMC 15912. To adjust the strain’s culture conditions, SSF was employed. The highest amount of xylanase was produced after 6 days at pH 7.0 and 30 °C when ammonium chloride was used as a nitrogen source. To isolate the xylanase, Trilite MC 08 cation exchange resin and Sephacryl S 200 columns were used for purification. Maximum activity of the purified xylanase was determined at 45 °C and pH 6.0. For the pure xylanase, the Km and Vmax were determined. In different degrees, xylanase activity was inhibited by NaCl, KCl, CaCl_2_, MgSO_4_, MnSO_4_, ZnSO_4_, FeSO_4_, CuSO_4_, NiSO_4_, SDS, and EDTA. Various immobilization techniques are required to generate recoverable, and reusable immobilized xylanase for a range of commercial applications across multiple areas of contemporary biotechnology.

### Future perspectives

This study’s findings underscore the potential of *A. krugeri* AUMC 15912 as a viable source of xylanase for industrial uses. Subsequent investigations should concentrate on many crucial domains to enhance the efficacy of this enzyme:

1. Enzyme immobilization and reusability
  Formulating economical immobilization methods will facilitate the recovery and reutilization of xylanase in continuous operations, hence decreasing operational expenses and enhancing sustainability.
2. Scale-up and bioprocess optimization
  Scaling up from laboratory-scale solid-state fermentation to pilot and industrial levels necessitates the optimization of factors like aeration, moisture regulation, and reactor design to sustain elevated enzyme production.
3. Genetic and metabolic engineering
  Advanced molecular techniques, including as CRISPR/Cas systems and omics-based methodologies, can be utilized to improve xylanase production, thermostability, and inhibitor tolerance, hence rendering the enzyme more appropriate for demanding industrial environments.
4. Integration into biorefinery platforms
  Integrating xylanase into biorefinery systems enhances the effective transformation of lignocellulosic biomass into biofuels, biochemicals, and value-added products, hence supporting circular economy objectives.
5. Exploration of synergistic enzyme cocktails
  The integration of xylanase with cellulases, pectinases, and other hemicellulases may enhance the saccharification efficiency of intricate substrates, hence improving overall process economics.
6. Industrial application trials
  Evaluating the enzyme in practical applications such as pulp and paper manufacturing, baking, juice clarifying, and animal feed supplementation will confirm its commercial feasibility and reveal potential enhancements.
7. Environmental and economic assessments
  Life cycle analysis and techno-economic evaluations must be performed to evaluate the sustainability and cost-effectiveness of large-scale xylanase manufacturing from agro-industrial residues such as sugarcane bagasse.
  Subsequent research should investigate the incorporation of xylanase synthesis inside biorefinery systems, facilitating the concurrent generation of co-products such as bioethanol and biochemicals. Integrated systems can enhance process economics and diminish environmental consequences, rendering xylanase production both commercially viable and environmentally responsible.

## Data Availability

The dataset generated and/or analyzed during the current study is available in the GenBank: [*Aspergillus krugeri strain AUMC 15912 internal transcribed spacer 1, p - Nucleotide - NCBI*] (https://www.ncbi.nlm.nih.gov/nuccore/OR393574.1); accession number OR393574.
